# Pathogenesis and treatment of Sjogren’s syndrome: Review and update

**DOI:** 10.3389/fimmu.2023.1127417

**Published:** 2023-02-02

**Authors:** Qipeng Zhan, Jianan Zhang, Yubin Lin, Wenjing Chen, Xinzou Fan, Dunfang Zhang

**Affiliations:** State Key Laboratory of Biotherapy and Cancer Center, Department of Biotherapy, Collaborative Innovation Center of Biotherapy, West China Hospital, Sichuan University, Chengdu, Sichuan, China

**Keywords:** sjogren's syndrome, foxp3 + treg, th17 cells, JAK pathway, tumor necrosis factor, interferon

## Abstract

Sjogren’s syndrome (SS) is a chronic autoimmune disease accompanied by multiple lesions. The main manifestations include dryness of the mouth and eyes, along with systemic complications (e.g., pulmonary disease, kidney injury, and lymphoma). In this review, we highlight that IFNs, Th17 cell-related cytokines (IL-17 and IL-23), and B cell-related cytokines (TNF and BAFF) are crucial for the pathogenesis of SS. We also summarize the advances in experimental treatment strategies, including targeting Treg/Th17, mesenchymal stem cell treatment, targeting BAFF, inhibiting JAK pathway, et al. Similar to that of SLE, RA, and MS, biotherapeutic strategies of SS consist of neutralizing antibodies and inflammation-related receptor blockers targeting proinflammatory signaling pathways. However, clinical research on SS therapy is comparatively rare. Moreover, the differences in the curative effects of immunotherapies among SS and other autoimmune diseases are not fully understood. We emphasize that targeted drugs, low-side-effect drugs, and combination therapies should be the focus of future research.

## 1 Introduction

Sjogren’s syndrome (SS) is a chronic autoimmune disease associated with functional disorders of the exocrine glands (e.g., parotid and lacrimal glands) and extraglandular manifestations. In 1892, JH Mikulicz reported the first case of SS. In 1933, the Danish ophthalmologist Sjogren reported on 19 female patients with dryness of the mouth and eyes, 13 of whom had rheumatoid arthritis (RA). To distinguish this ailment from xerophthalmia (vitamin A-deficiency-related dryness of the eyes), Sjogren defined the syndrome as keratoconjunctivitis sicca. KJ Bloch presented the clinical features of the currently recognized syndrome and introduced primary Sjogren’s syndrome and secondary Sjogren’s syndrome, which presents without and with an independent connective tissue disease (CTD), respectively (1).

According to a worldwide epidemiological study based on PubMed and Embase data, the incidence rate of SS is 6.92 per 100 000 person-years and the prevalence rate is 60.82 cases per 100 000 inhabitants, or 1 case per 1644 persons. Moreover, the age of patients peaks at 56. In the last 15 years, the disease has affected females more than males (2). Patients with SS experience an enduring and intolerable pain with multiple physical symptoms, such as dental caries, vaginal dryness, and arthralgia (3).

Given the immense social and economic burden caused by SS, we aimed in this review to characterize the current paradigm of the pathogenesis and treatment of SS to motivate and inform the development of efficient treatment strategies, particularly immunological treatments.

## 2 Brief review of clinical manifestations

**Figure 1:**
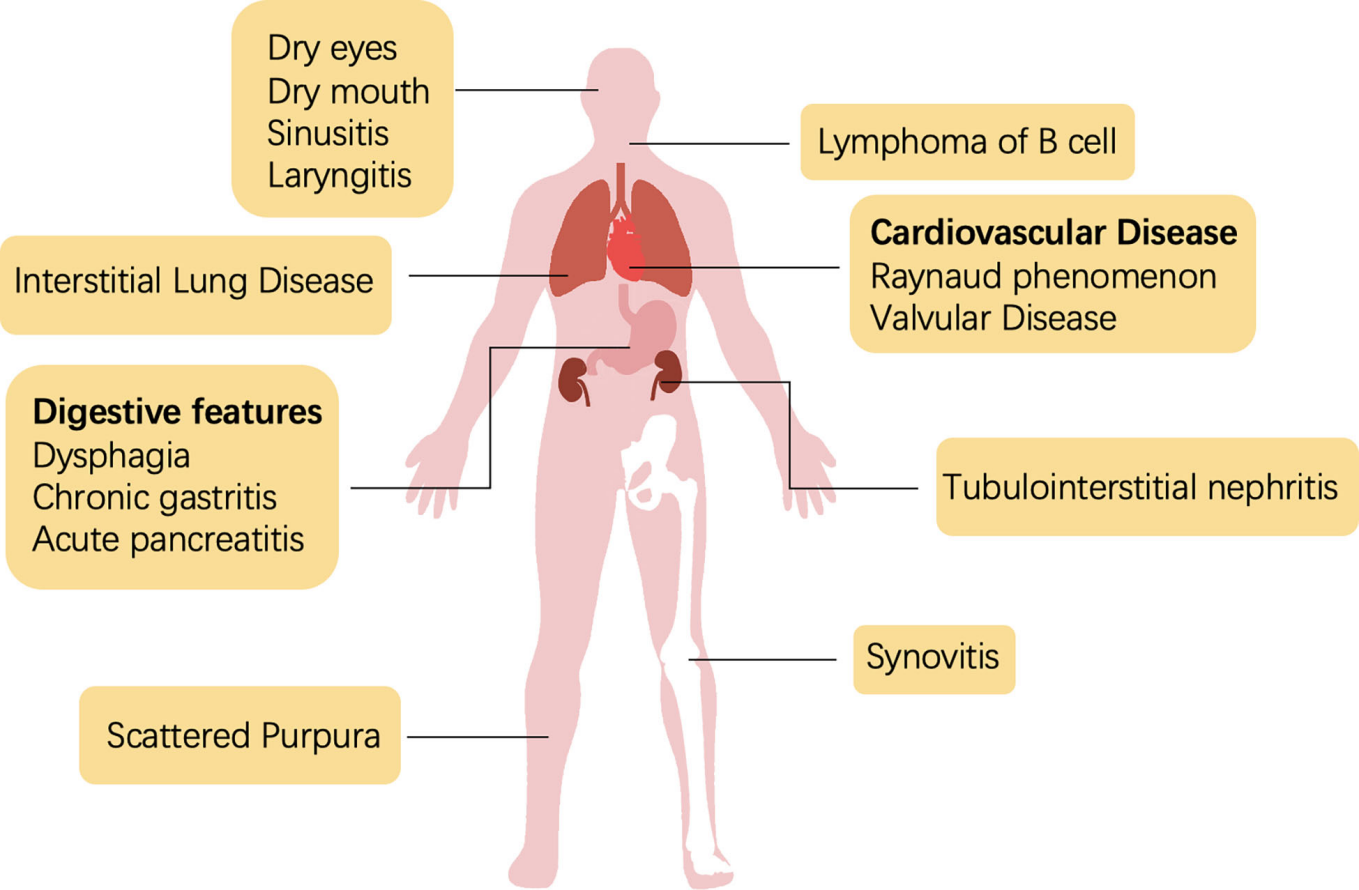
Typical glandular and extraglandular manifestations of SS.

Sjogren’s syndrome is a systemic disease with heterogeneous manifestations that involve disorders or damage to the tissues of the exocrine glands (**Figure 1**). The diagnosis of SS is a multistep process, including the evaluation of oral and ocular dryness, detection of anti-SSA/Ro and anti-SSB/La antibodies, and glandular biopsy. Dryness of the eyes and mouth, which is caused by the dysfunction of salivary and lacrimal glands, is the most salient and common clinical symptom. Severe sicca symptoms of the eyes and mouth profoundly impede quality of life. An aqueous-deficient mouth has a severe effect on oral health and is associated with an increased risk of developing caries (4). A recent study reported that the oral microbiome of patients with SS who have salivary hypofunction was under stress and dysregulated; *Veillonella parvula* is a potential biomarker of Sjogren’s syndrome (5).

Because SS presents with multiple extraglandular manifestations (**Figure 1**), the European Alliance of Associations for Rheumatology (EULAR) developed The EULAR SS disease activity index (ESSDAI) to assess disease activity in patients with sicca symptoms and simplify diagnosis. ESSDAI evaluates the severity of disease within 12 clinical domains (i.e., constitutional, lymphadenopathy, glandular, articular, cutaneous, pulmonary, renal, muscular, peripheral nervous system, central nervous system, hematological, biological), and it aims to obtain a standardized evaluation in clinical trials and practice (6).

The involvement of the nervous system was first reported in the 1980s (7). Several neurological diseases have since been associated with SS (8–12), which indicates the importance of precise neurological diagnostic assessments. Interstitial lung disease (ILD) is the most frequent and severe pulmonary complication of SS and contributes substantially to morbidity and mortality. In an Italian cohort, approximately 20% of patients with comorbid SS presented with ILD, and approximately 10% presented with amyloidosis and primary lung lymphoma (13). Efficient clinical examination, including lung biopsy or screening of serological markers, could assist in the early diagnosis and intervention of SS-ILD (13). Unfortunately, no effective treatment strategy exists for SS-ILD (14). As an autoimmune disease, SS can also lead to synovitis and RA, with the latter causing structural damage. A previous study showed that the medication strategy of RA had some success in SS, but the best-performing regimen is unclear (15).

Renal complications have only been observed in less than 10% of patients with SS. Tubulointerstitial nephritis (TIN), caused by lymphocyte infiltration around the renal tubes, occurs in two-thirds of patients with SS and renal dysfunction (16, 17). However, the low prevalence of renal manifestations may be an artefact of the ineffective diagnosis of TIN (18). Non-Hodgkin’s lymphoma (NHL) is the most severe extraglandular complication of SS, with the B cell type being predominant (occurs in approximately 5% of SS cases) (19). Hypergammaglobulinemia or the aberrant expression of other antigens in the blood stimulate the expansion of rheumatoid factor-reactive B cells (20). Meanwhile, B lymphocyte-activating factor (BAFF) and germinal center (GC)-like structures amplify the activation of B cells (21, 22). Some studies have reported that SS-NHL is also associated with abnormal activation of nuclear factor kappa B (NF-κB) (23, 24). Additionally, a multicenter clinical study showed that more than a quarter of patients with SS presented systemic symptoms beyond the current ESSDAI classification, including cardiovascular; digestive; pulmonary; ear, nose, and throat (ENT); cutaneous; and urological features (25).

## 3 Pathogenesis

### 3.1 Brief introduction

In a 2013 review by G Nocturne and X Mariette of the pathogenesis of SS (26), three key steps were identified based on the initial genome-wide association study (GWAS): aberrant activation of the innate immune response, especially through the interferon (IFN) and NF-κB pathways, atypical recruitment to lymphoid follicles mediated by CXCR5, and T cell activation with ascending HLA susceptibility along the IL-12–IFN-γ axis. BAFF was considered to be vital in coordinating the innate and adaptive immune responses to the disease. They also highlighted the pathophysiological role of natural killer (NK) and epithelial cells as well as the dysfunction of the neuroendocrine system.

Mavragani et al. reviewed the treatment strategies and molecular targets of the innate and adaptive immunity pathways (27). Regarding the regulation of innate immunity, previous research focused on inhibiting the production of proinflammatory factors, such as IL-1, IL-6, and tumor necrosis factor-α (TNF-α), which has proven to be effective in other autoimmune diseases. IFN-associated pathway inhibitors were another research topic of interest. For example, downregulating the expression of the primary dendritic cell surface receptor ILT7 to reduce TLR7/9-mediated IFN production was considered a potential treatment route. Regarding adaptive immunity, previous research focused on antigen presentation, co-stimulation, B-cell activation, T-cell proliferation, and germinal center formation. Overall, most strategies were aimed at regulating aberrant inflammation.

### 3.2 IFN

IFN is an immunoregulatory protein that promotes innate and acquired immunity and antiviral activation. IFN is categorized into three types based on structure and origin, i.e., I, II, and III. In 1981, researchers detected type-I IFN in the blood of patients with autoimmune disease, and linked its expression to the clinical manifestations (28). IFN-I plays an important role in the progression of SS by promoting the activity of immune cells, such as NK cells, CD8^+^ T cells, and even macrophages. In addition, dendritic cells, the main producers of IFN-I, were observed in the salivary glands of patients with SS, which suggests a role for IFN-I in the formation of salivary gland lesions (29, 30) (31, 32).

IFN activates the overexpression of canonical interferon-stimulated genes (ISGs) through the Janus kinase (JAK)-STAT signaling pathway, which is defined as the “interferon signature.” IFN phosphorylates STAT1, STAT2, STAT3, and STAT5, which activate downstream signals leading to the activation of immune cells (33–35). This signature in gene expression is considered a biomarker of autoimmune diseases (36).

Under physiological conditions, *in vitro*-derived pathogens or *in vivo*-derived apoptotic cells can trigger a rapid innate immune response through pattern recognition receptors (PRRs), including TLRs, RLRs, and NLRs (37). PRRs can recognize nucleic acids and induce the production of numerous proinflammatory cytokines and type I IFNs; thus, aberrant activation of the self-antigen recognition Toll-like receptor (TLR) leads to the development of autoimmune disease (38).

Some studies have reported the enhanced expression of the cell adhesion molecules VACM-1, ICAM-1, and programmed death ligand-1 (PD-L1) in patients with SS (39–41). The aberrant expression of these cytokines is mediated by IFN-I and IFN-II through the JAK-STAT pathway (42, 43). A recent study used reactive oxygen species (ROS) and N-acetylcysteine (NAC) to induce or block the expression of ICAM-1 and PD-L1 and revealed that the IFN signature that regulates the expression of ICAM-1 and PD-L1 in SS was related to oxidative stress (43–45).

Several recent studies have suggested that IFN-III contributes to SS. Type III IFNs, which consist of IFN-λ1, IFN-λ2, IFN-λ3, and IFN-λ4, are mainly produced by plasmacytoid dendritic cells (pDCs) (46, 47). pDCs respond to the secretion of IFN-III and show improved survival under stimulation with IFN-III *in vitro*. IFN-III enhances the production of IFN-I and TNF-α in pDCs and promotes the expression of CD80 and CD86, which contribute to the maturation of pDCs (35). IFN-III regulates the immune response by upregulating the polarization of Th1 and CD8^+^ T cells and downregulating Th2 cytokines and Tregs (48).

### 3.3 Genome loci associated with SS

Etiological research has revealed the pathogenesis of SS at the genomic level. A GWAS of autoimmune diseases identified an association between HLA regions and SS, including HLA-DR, HLA-DQB1, and HLA-DQA1 (**Table 1**). The allele with the strongest association was HLA-DQB1^*^0201 (*P*_meta_ = 1.38 × 10^–95^). All HLA alleles were correlated with the expression of rs115575857. In addition, six non-HLA regions that surpassed the suggestive threshold (*P*_meta_ < 5 × 10^–5^) were also shown to be involved in SS, including *IRF5*, *STAT4*, *BLK*, *IL-12A*, *TNIP1*, and *CXCR5*, with the expression of *IRF5* and *STAT4* being the most significant contributors after HLA regions (**Table 1**) **(** 49).

**Table 1:** Genome loci associated with SS.

*Gene loci*	SNP	Encoding protein	Effect pathway	P value of Meta-analysis
*HLA*	rs112357081	MHC-II	Antigen presentation	7.65 × 10^-114^ ~ 1.37 × 10^-85^
	rs3135394			
	rs115575857			
	rs3129716			
	rs116232857			
	rs9271588			
*IRF5*	rs3757387	Interferon regulatory factor 5	Activate IFN	2.73 × 10^-19^ ~ 3.20 × 10^-6^
	rs4728142			
	rs17339836			
	rs17338998			
	rs10954213			
*IL-12A*	rs485497 rs583911	Interleukin-12 α	T-cell-independent production of IFN	1.17 × 10^-10^ ~ 9.88 × 10^–9^
*BLK*	rs2736345	B lymphocyte kinase	Activate B cells	4.97 × 10^-10^ ~ 7.96 × 10^-8^
	rs2729935			
	rs6998387			
*CXCR5*	rs7119038	CXC chemokine receptor 5	Mediate migration of B cells	1.10 × 10^-8^ ~ 6.82 × 10^-8^
	rs4936443			
*TNIP1*	rs6579837	TNFAIP3-interacting protein 1	Regulate NF-κB	3.30 × 10^-8^ ~ 5.32 × 10^-7^
	rs7732451			
*STAT4*	rs10553577	signal transducer and activator of transcription 4	Regulate differentiation of helper T cells	6.80 × 10^-15^ ~ 9.45 × 10^-9^
	rs13426947			

Various genome loci were reported to be associated with the pathogenesis of SS. Both HLA regions (HLA-DR, HLA-DQB1, HLA-DQA1) and non-HLA regions (*IRF5, STAT4, BLK, IL-12A, TNIP1*, and *CXCR5*) were established as risk loci.

### 3.4 Type 17 helper T (Th17) cells/IL-17

Th17 cells are distinct from Th1/Th2 cells and regulate immune responses independently (50, 51). Th17 cells polarize naïve T cells after stimulation by TGF-β and IL-6 from antigen-presenting cells (APCs). IL-1β secreted from the ductal epithelium and IL-23 secreted from DCs also participate in Th17 cell polarization. IL-17 and IL-22 are produced by and are the main effective cytokines of Th17 cells. Th17 cells mediate inflammation by producing the proinflammatory cytokines TNF-α and IL-6 (52). Previous research revealed that IL-17/IL-23 expression was enhanced in mouse models with SS, indicating that Th17 participated in lymphocytic infiltration of salivary glands and contributed to lesion formation (53, 54). Another study showed that IL-22, IL-23, and IL-17 were increased in the peripheral blood of patients with SS, both at the protein and mRNA levels. Notably, in addition to Th17 cells, NKp44^+^ NK cells can also produce IL-17 in patients with SS (55). Besides, Th17 cells are potent inducers of matrix metalloproteinase 1 (MMP1) and MMP3 (56), and a study has shown that SS is related to disorders of MMP3/tissue inhibitor of metalloproteinase 1 (TIMP1) and MMP9/TIMP1 ratios (57).

Both Treg and Th17 cells can be induced by TGF-β from activated T cells, indicating that there might be a balance between these opposing inflammation-related cells. An imbalance in the Th17/Treg ratio has been reported in several other autoimmune diseases, including inflammatory bowel disease (IBD) (58, 59), autoimmune thyroid disease (AITD) (60), psoriasis (61), multiple sclerosis (62), and RA (63, 64). In these diseases, function and stability of Treg cells are impaired, and the aberrant induction and proliferation of Th17 cells result in the activation of other immune cells, which then drive an acute autoimmune response. Metabolic pathways play an important role in the regulation of the Th17-Treg cell network. Th17 cells are glycolysis-dependent; thus, by inhibiting the mammalian target of rapamycin (mTOR) pathway with rapamycin, glycolysis is inhibited and the polarization of Th17 cells is decreased, whereas the expression of Treg cells is increased (65). Tregs tend to increase glycolysis and enhance fatty acid oxidation, while Th17 cells rely on fatty acid synthesis (66). However, current metabolic models of Th17/Treg cell regulation through the glycolysis pathway are inconclusive. Fortunately, Compass (67), a powerful algorithm based on scRNA-sequencing and flux balance analysis, was recently produced to predict the relationship between cellular metabolic states and pathogenicity, and has already been utilized in research on the Th17-Treg network.

Type 17 follicular helper T (Tfh17) and IL-17-producing B (B17) cells also contribute to IL-17 production. A recent study revealed that the number of IL-17-producing cells increased in the peripheral blood and spleen of NOD/ShiLtJ mice with STZ-induced type I diabetes and SS. Surprisingly, the infiltration of IL-17-producing cells in the salivary glands increased in metabolically disordered murine models, and was also associated with greater severity of SS (68). It was subsequently found that the aberrant expression of IL-17 induced by metabolic abnormalities contributed to cell lesions and inhibition of tissue recovery in the salivary glands of patients with SS. Furthermore, retinoic A deficiency can exacerbate the imbalance in the Th17/Treg ratio in patients with SS (69).

### 3.5 TNF/BAFF

TNF-α is predominantly produced by macrophages and T cells in two forms: soluble TNF-α (sTNF-α) and transmembrane TNF-α (Tm TNF-α). sTNF-α is an effective regulator of inflammation and autoimmune diseases (70). TNF-α can bind with TNFR1 or TNFR2 and mediate inflammation by activating the NF-kB pathway and mitogen-activated protein kinases (MAPKs) (71).

BAFF (or BLyS) is a member of the TNF family that plays a vital role in B cell survival. Usually, BAFF is produced by neutrophils, macrophages, monocytes, DCs, and follicular DCs (72). Increased levels of IFN-I, IFN-γ, IL-10, and G-CSF can induce the expression of BAFF, while TLR3, TLR4, or TLR9 participate in BAFF production (73, 74). B cells express BAFF receptors (BAFF-R or BR3), as well as TACI and BCMA. A previous study reported that BAFF binds to BAFF-R and enhances the conversion of NF-κB2/p100 to p52 (75). Additionally, BAFF binds to BAFF-R, activating the PI3K-AKT1 pathway, which regulates the activation of myeloid cell leukemia sequence 1 (MCL1) and inhibits BCL-2-interacting mediator of cell death (BIM). TNF receptor-associated factor 3 (TRAF3) and TRAF2 are intracellular signaling molecules that bind to BAFF-R or TACI. BAFF-R interacts with BAFF and recruits TRAF3, resulting in the degradation of TRAF3 and inhibition of the NF-κB pathway. Nevertheless, the binding of TACI and BAFF results in the recruitment of TRAF2 or TRAF6 and promotes the activation of the NF-κB pathway (72).

BAFF participates in the pathogenesis of various autoimmune diseases, including RA (76), SLE (77), Graves’ disease (78), and anti-GBM disease (79). Overexpression of BAFF elevates MHC-II expression, enhances lymphocytic infiltration, and increases the number of germinal center (GC)-like structures in SS murine models (80). Increased GC-like structures are associated with enhanced production of rheumatoid factor, anti-RO/SSA, anti-La/SSB, and IgG in patients with SS (81). However, another study claimed that BAFF is unable to mediate the differentiation of B cells from GCs, which suggests the involvement of the inhibitory BAFF-TACI pathway (80). Furthermore, BAFF stimulates monocyte through binding with BAFF-R and fosters the production of IL-6, which induces the aberrant production of IgG from B cells in SS (82).

### 3.6 Wingless/integrated signaling pathway

The Wnt signaling pathway is involved in several biological processes, including cellular migration, proliferation, differentiation, apoptosis, tissue homeostasis and regeneration, and stem cell self-renewal (83). Dysregulation of the Wnt/β-catenin pathway plays a vital role in the pathogenesis of many cancers and autoimmune diseases (84, 85).

The role of the Wnt signaling pathway in T cell differentiation and immune regulation has been elucidated (86–88). The disorder of Wnt signaling inhibitors in autoimmune diseases was also noticed. Proinflammatory cytokines promote bone damage by fostering the production of Wnt signaling inhibitors, including secreted frizzled-related, Wnt inhibitory factor 1, sclerostin, and Dickkopf (DKK) family proteins (89–91). However, it was found that the role of DKK-1 is different in various autoimmune diseases (92). In a clinical study of 98 SS patients and 165 healthy volunteers, three Wnt/β-catenin signaling pathway-related genes, LRP5, FRZB, and ADIPOQ, were shown to increase the risk of SS, although the biological functions of these genes have not yet been established (91). It implicates Wnt pathway might be involved in the pathogenesis of SS. However, not all studies support this idea. A clinical study reported that serum Dkk-1 and sclerostin levels were decreased in SS and SLE, and the Wnt1 and Wnt3a levels had no significant changes (93).

### 3.7 IL-33/ST-2

The IL-33-ST2 axis participates in the pathogenesis of SS by promoting transcriptional activation of CD86 and CCL2 in salivary epithelial cells and activation of the NF-κB pathway. IL-33, combined with IL-12 and IL-23, participates in the production of CD4^+^ T cell-derived and NK/NKT-derived IFN-γ (89, 94). An increase in serum levels of IL-33 and ST2 has been reported in patients with SS (95). IL-33 is a member of the IL-1 family and ST2 is one important member of IL-1 receptor family (90). IL-33 induces phosphorylation of the NF-κB pathway and activates MAP kinases by interacting with ST2 to stimulate downstream Th2-related immune responses. A recent review described IL-33 as an alarmin, that is, a DAMP. Local increases in IL-33 expression can induce an immune response and result in organ lesions (91). The IL-33-ST2 axis is a novel mode in the pathogenesis of SS and a potential therapeutic target in related salivary gland disorders.

## 4 Experimental therapeutic strategies of SS

### 4.1 Targeting the Treg/Th17

The Treg/Th17 plays a crucial role in the pathogenesis of autoimmune diseases. Various drugs have been designed to target the molecular mechanisms involved in the polarization and activation of the Treg/Th17, including IL-17-related molecules (IL-17, IL-23), transcription factors (RORγt, STAT3, Foxp3, and FoxO1), and intracellular signaling pathways (ROCK and MAPK) (96). However, compared to RA, SLE, IBD, and psoriasis, clinical or preclinical drugs for SS are rare. Nevertheless, previous pharmacological exploration and clinical trials have provided promising results for SS therapy.

**Figure 2:**
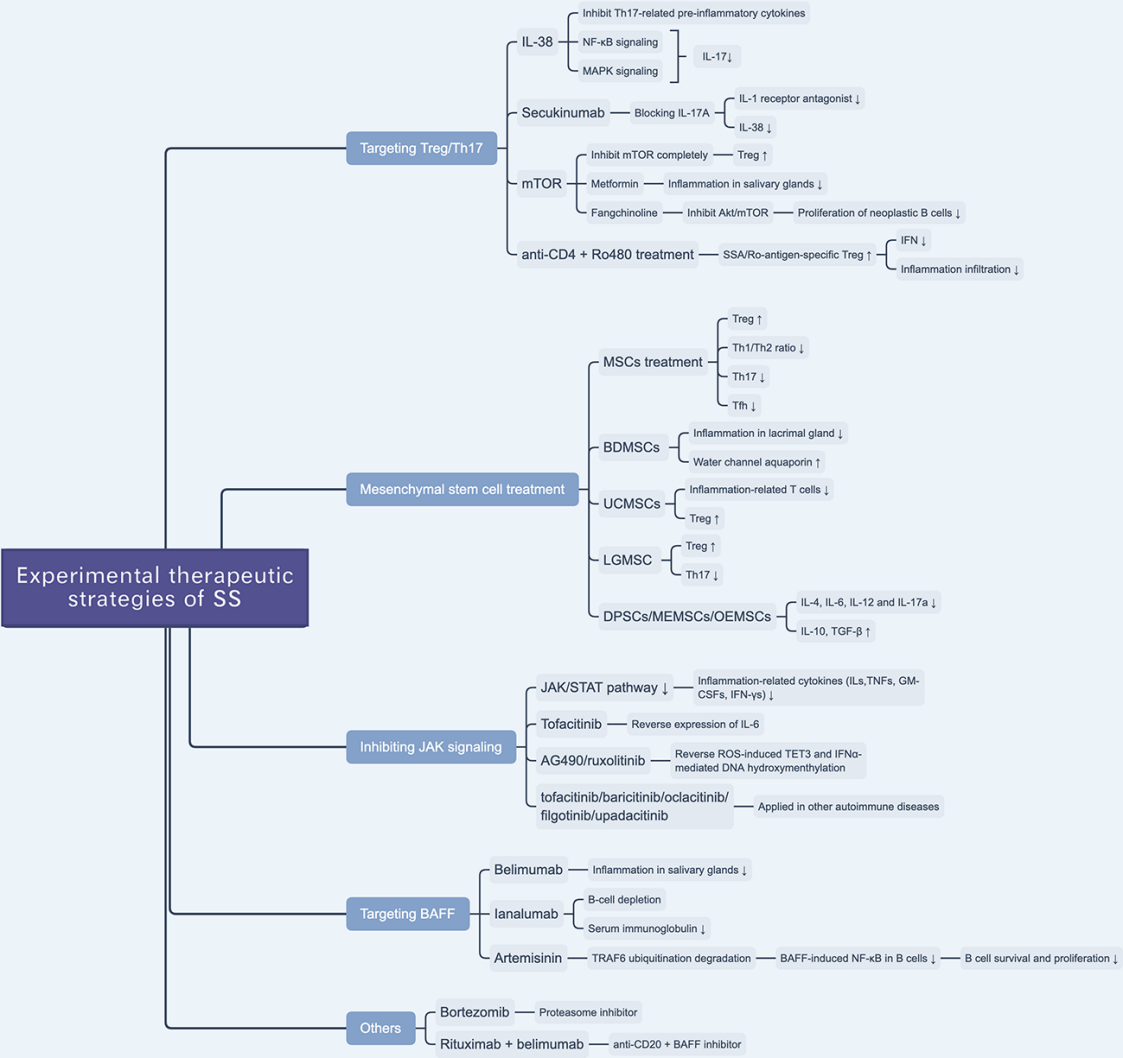
Overview of novel experimental therapeutic strategies of SS. There are various experimental drugs (e.g. JAK inhibitors) or schemes (e.g. targeting Treg/Th17 and BAFF) being investigated for SS treatment. Besides, mesenchymal stem cell treatment is reported to be a promising scheme to treat SS.

IL-38—a member of the IL-1 family and, which was named as IL-1F10 (97)—inhibits the secretion of Th17 cell-related cytokines, including IL-6, IL-8, IL-17, IL-22, and IL-23, by binding with IL-36 receptors (98). A previous clinical study reported a selective anti-IL-17A mAb, secukinumab, that can alleviate the symptoms of psoriasis by blocking the expression of IL-17A, while that of IL-1 receptor antagonist (IL-1ra) and IL-38 was downregulated (**Figure 2**) **(** 99). A previous study tested the effect of IL-38 treatment on Th17 cell activity and found that the expression levels of IL-17 and IL-23 were decreased in a murine model of SS; IL-38 inhibited IL-17 expression through the NF-κB and MAPK signaling pathways(**Figure 2**). They also found that IL-17 can upregulate the expression of IL-38 (54). This hints at a potential approach for the treatment of SS.

mTOR, a member of the phosphoinositol 3-kinase (PI3K) family, is an atypical serine/threonine kinase that plays a vital role in cellular metabolism and activity (100). mTOR likely prevents anergy induction by IL-2 expression in T cells. A previous study found that by blocking mTOR with rapamycin, the cell cycle of clonal T cells was inhibited, while it induced cell anergy even with costimulations (101). In the process of naïve T cell differentiation, mTOR mediates the transformation to Th17 or Treg cells by altering the sensitivity of T cells to TGF-β, which influences the effects of STAT3 signaling (102). The inhibition of different mTOR complexes (including mTORC1 and mTORC2) would activate different pathways of polarization to Th17 cells, whereas a complete inhibition of mTOR can promote polarization to Treg cells (103). mTOR plays a role in Th17/Treg balance, given that mTOR inhibitors interfere with the Th17/Treg ratio, which suggests a potential therapeutic target to ameliorate glandular lesions in patients with SS. Given the anti-inflammatory and immunomodulatory effects of metformin—an AMPK-dependent mTOR-STAT3 inhibitor—researchers examined its therapeutic effect in SS murine models and found that it ameliorated inflammation in the salivary glands and, based on flow cytometry, regulated the Th17/Treg ratio (**Figure 2**) **(** 104). Yu et al. reported that an alkaloid extracted from the traditional Chinese herbal medicine Stephania tetrandra S. Moore, fangchinoline, can be used to treat SS by inhibiting the Akt/mTOR pathway, which inhibits the proliferation of neoplastic B lymphocytes (**Figure 2**) **(** 105).

More than that, a recent study reported that SSA/Ro-antigen-specific Treg cells can downregulate the production of CD4^+^ T cell-derived IFN-γ and suppress inflammatory infiltration of the salivary gland (106). Researchers reported that the combination treatment with anti-CD4 mAb and autoantigen-specific peptide Ro480 induces SSA/Ro-antigen-specific Treg cells *in vivo* and suppresses CD4^+^ T cell-related IFN-γ production in salivary glands, providing a potential novel immunotherapeutic strategy for the treatment of SS (**Figure 2**) **(** 106).

### 4.2 Mesenchymal stem cell treatment

Mesenchymal stem cells (MSCs) exert immunomodulatory effects on both adaptive and innate pathways. MSC can manipulate the balance between suppressive Treg cells and inflammatory T helper cells (Th1, Th2, Th17, and Tfh) and ameliorate inflammatory infiltration in the salivary glands (107, 108).

Xu et al. revealed that immunomodulatory functions of MSCs are impaired in SS-like murine models, and allogeneic bone marrow mesenchymal stem cells (BMMSCs) infusion can suppress SS-like inflammation, showing therapeutic effects of BMMSCs on SS (109). Furthermore, they also elucidated that the stromal cell-derived factor-1(SDF-1)/C-X-C chemokine receptor 4(CXCR4) axis plays an important role in MSC migration and restoration of salivary glands. More than that, they also treated twenty-four SS patients with umbilical cord-MSCs (UCMSCs), and all patients showed alleviation of SS symptoms and well tolerance of allogeneic UCMSCs (**Figure 2**) **(** 109).

Zoukhri et al. revealed that a biotherapeutic strategy involving bone-derived MSCs (BDMSCs) alleviated lacrimal glandular manifestation in a SS murine model by inhibiting inflammation and promoting the expression and activation of water channel aquaporin 5 (**Figure 2**) **(** 110). Li et al. verified the immunomodulatory effects of UCMSCs and found that UCMSCs induced CD4^+^FoxP3^+^ Treg cells *in vitro* and caused anergy of inflammation-related T cells *in vivo*, accompanied by an increase in Treg cells (111). Hua et al. assessed the effects of labial gland-derived MSCs (LGMSCs) and their exosomes on SS, and found that they ameliorated salivary gland inflammatory infiltration by inhibiting the polarization of Th17 cells and promoting the proliferation of Treg cells (**Figure 2**) **(** 112). Furthermore, dental pulp stem cells (DPSC) (113), murine embryonic MSCs (MEMSCs) (114), and olfactory ecto-MSCs (OEMSCs) (115) can be used to treat SS by interfering with inflammation-related cytokines (IL-4, IL-6, IL-12, and IL-17a) and suppressive cytokines (IL-10 and TGF-β) (**Figure 2**) **(** 116).

### 4.3 Inhibiting JAK pathway

JAK enzymes are involved in the JAK/STAT pathway through the phosphorylation of STAT, which leads to the activation of signals transferred to the nucleus. The JAK family consists of four members, JAK1, JAK2, JAK3, and TYK2 (117). Membrane receptor subunits usually bind to a specific JAK. For example, JAK3 can only selectively binds to the γc chain, which is a common receptor chain of IL-2, IL-4, IL-9, IL-15, and IL-21 (118).

The JAK/STAT pathway regulates the production of ILs, TNFs, GM-CSFs, and IFN-γs, which are associated with inflammation and autoimmunity (119). The JAK inhibitors tofacitinib (120), baricitinib (120), oclacitinib (121), filgotinib (122), and upadacitinib (123) have been applied in the treatment of autoimmune diseases (**Figure 2**). Renaudineau et al. reported that AG490 and ruxolitinib, two JAK1/2 inhibitors, can reverse ROS-induced production of ten-eleven translocation 3 (TET3) and IFNα-mediated DNA hydroxymethylation and could potentially treat SS (**Table 2**) **(** 45, 132). Tofacitinib is also a candidate drug for SS, given that it can reverse the expression of IL-6 in ATG5-deficient 3D-acini, which leads to the inhibition of inflammation (**Table 2**) **(** 133).

**Table 2:** Experimental Biotherapeutic Drugs of SS.

*Drug*	*Effect target*	*Mechanism*	Feasibility^*^	*References*
*Rituximab*	CD20	Induce ADCC and CDC	-	(124)
*Belimumab*	BAFF	Inhibit the combination of BLyS and B cells	+	(125)
*Artemisinin*	BAFF	Downregulate the BAFF-induced NF-κB activity	+	(126)
*Iscalimab*	CD40	Inhibit the combination of CD40 and CD40L	–	(127)
*Tocilizumab*	IL-6 receptor	Block IL-6R	-	(128)
*Ianalumab*	BAFF receptor	Block BAFF receptor	+	(129, 130)
*Abatacept*	CD80&CD86	Inhibit activation of T cells	+	(131)
*Ruxotinib*	JAK/STAT pathway	Inhibit IFN and ROS related DNA hydroxymethlation	*	(45, 132)
*AG490*	JAK/STAT pathway	Inhibit IFN and ROS related DNA hydroxymethlation	*	(45, 132)
*Tofacitinib*	JAK/STAT pathway	Decrease expression of IL-6	*	(133)
*Bortezomib*	Proteasome pathway	Inhibit activation and nuclear translocation of NF-κB	+	(134)

* Experimental effects on treating SS. “-” means none or low effect. “+” means promising effect. “*” means unclear.

### 4.4 Targeting BAFF

Belimumab is an anti-BAFF monoclonal antibody and a potential biotherapeutic drug for SLE and SS (**Table 2**) **(** 125, 135). A bi-centric clinical trial reported that, after a 28-week regimen of belimumab (10 mg/kg, at weeks 0, 2, 4, and then every 4 weeks), 18 out of 30 patients achieved two of five primary endpoints. The mean and standard deviation of ESSDAI and EULAR Sjogren’s Syndrome Patients Reported Index (ESSPRI) were both reduced (**Figure 2**) **(** 125).

Ianalumab is a BAFF-blocking monoclonal antibody that leads to B-cell depletion (**Figure 2**). A previous clinical study found that, in SS, ianalumab reduced the ESSDAI, ESSPRI, and serum immunoglobulin levels (**Table 2**) **(** 129).

Besides, Zheng et al. reported a Chinese herb-derived drug, Artemisinin (ART), which is used to treat chloroquine-resistant malaria originally, has immunosuppressive effects in the SS-like murine model (**Figure 2**) (126). The study demonstrated that ART downregulates BAFF-induced NF-κB activity in B cells through targeting TRAF6 ubiquitination, which results in the inhibition of B cell survival and proliferation. Therefore, the levels of B lymphocyte-related immunoglobulin and autoantibody in the SS-like murine model were attenuated and lymphocytic infiltration in the salivary gland was ameliorated (**Table 2**) (126).

### 4.5 Others

Bortezomib is a proteasome inhibitor used in the treatment of multiple myeloma (**Table 2**). A Mexican case report described a female patient that suffered from SS for 16 years and presented with sicca complex, extreme fatigue, Raynaud phenomenon, generalized arthralgia, and heavy headaches. After ineffective conventional glucocorticoid and rituximab therapy, doctors administered an experimental regimen of bortezomib at a dose of 1.3 mg/m^2^ (2.0 mg/dose) at days 1, 4, 8, 11, 22, 29, 36, 43, 50, and 57. Surprisingly, the patient’s headaches and fatigue were resolved after three months, and serum globulin levels and viscosity decreased significantly (**Table 2**) **(** 134). However, the efficacy and safety of bortezomib for the treatment of SS are still unconfirmed (**Figure 2**).

Rituximab—a chimeric monoclonal anti-CD20 antibody—has been reported to induce B-cell depletion and has been used to treat autoimmune diseases (136, 137). It has also been used to treat SS over the last 20 years, but with limited clinical efficacy (**Table 2**) **(** 124, 138). Researchers have investigated a combination therapy with the anti-BAFF and anti-CD20 [NCT02631538]—belimumab and rituximab. The results of the clinical trial provide evidence that simultaneous targeting of the BAFF axis and B cells is a promising treatment strategy for SS (**Figure 2**) **(** 139). The drugs in development for SS treatment are summarized in **Table 2**.

Various biotherapeutic drugs have been used to treat SS experimentally. Some of them have been used to treat other autoimmune diseases (e.g. Rituximab), and the others are novel drugs targeting inflammation-related signaling pathways (e.g. AG490). However, not all of them have prospective effects on treating SS (e.g. Iscalimab, Tocilizumab).

## 5 Conclusion & discussion

As a systemic autoimmune disease, SS causes multiple organ lesions, especially in salivary and lacrimal glands, which limits endocrine function. Besides focal inflammation in the salivary gland, acinar atrophy, duct dilation, and fibrosis are commonly observed in SS patients. Due to a high disease specificity and limited invasiveness, labial salivary gland biopsy is widely accepted as the best method to diagnose SS currently (140). Lymphocytic infiltration around the striated ducts in salivary glands, or so-called periductal foci, is a critical hallmark for the diagnosis of SS (141). Since adipose tissue replacement in the salivary gland is related to the stages of SS, and adipocytes are detected in IL-6-rich regions, detecting the degree of adipose tissue replacement provides aid to improve diagnosis accuracy (142). In addition, comorbidities, such as secondary pulmonary disease, kidney injury, and lymphoma, further reduce the quality of life of patients. The pathogenesis of SS is characterized by the production of inflammatory cytokines and lymphocyte infiltration. IFN and IL-17/Il-23 play pivotal roles in the formation of inflammatory lesions, and B cells are crucial for infiltrative injury. Th17, B, and dendritic cells play critical roles in the aberrant regulation of the immune system.

Similar to other autoimmune diseases, such as SLE, RA, and psoriasis, the traditional therapeutic strategy for SS is disease-modifying antirheumatic drugs (DMARDs), such as glucocorticoids, while novel biotherapeutic approaches take advantage of neutralizing antibodies and inflammation-related receptor blockers. Compared with the traditional strategy, this new scheme is more targeted, which can promote safety and efficacy. Although considerable progress has recently been made in the treatment of SS, disease-specific drugs are rare. Many SS drugs are currently undergoing clinical trials. As Th17 and B cells play important roles in the pathogenesis of SS, the targeting of Th17 cell- and B cell-related signaling pathways and molecular events has drawn increasing attention.

Research on SS therapies is limited by a lack of systematic clinical trials compared with other autoimmune diseases, such as SLE, RA, and MS. Many potential therapeutic targets have been identified in the pathogenesis of SS, and some targeted drugs have shown reasonable efficacy under experimental conditions *in vitro* or *in vivo*. Unfortunately, the translation of these drugs to clinical use is rare. Additionally, it is unclear why immune inhibitors lack pharmacological effect in SS compared to SLE, RA, and MS. Nevertheless, immune inhibitors can be used in the management of complications to improve the prognosis and quality of life of patients with SS. For example, BAFF receptor blockers not only prevent inflammatory lesions but also protect against B-cell lymphoma; however, such therapeutic strategies are rare. Combination therapies have shown some efficacy; however, inappropriate combinations of drugs may cause excessive inhibition of the immune system, resulting in unexpected complications, such as secondary infections. Thus, targeted and low-side-effect drugs should be the focus of future research.

## Author contributions

QZ drafted the manuscript. JZ, YL, WC and XF edited the manuscript. DZ supervised the work and edited the manuscript. All authors contributed to the article and approved the submitted version.
